# Winter Feedings

**Published:** 1891-01-17

**Authors:** 


### The Doctor's Armchair.

### WINTER FEEDING.

We have been called upon pretty often lately to help to feed other people. The severity of the season bids us give a little special consideration to the question of feeding ourselves. A man does not need to be much of a philosopher to understand that he may require different food when the thermometer stands at 28 deg., from what he needs when it stands at 82 deg. When science, which—and not law—is the "perfection of reason," shall have established her authority over every ordinary man's and woman's mind, there will be as much difference between the food of the summer and that of the winter, as there is between the temperature of the two periods. Everybody can understand that many persons among the poorer classes have died during the past few weeks from insufficient food; but very few will be prepared to believe, off-hand, that many persons among the better classes, during the same cold period, have died from improper food. And yet the latter is quite as true as the former. Life may be likened to a fire, which, if properly attended to, and intelligently "nursed," may be kept burning for a wonderfully long time. There are periods in the history of the fire of life when the clumsiest stoker with his insanest shovelings, can hardly put it out; and there are other periods when the embers are so dull and low that a single piece of coal put too hastily on, or put on in the wrong place, may extinguish the flame for ever.

Extreme cold has two effects upon people; if it be dry it acts as a tonic upon the young and strong, just as a fierce wind fans a strong flame and makes it ten times larger and stronger. But upon the aged and feeble the cold, even though it be dry, has a withering and blighting effect, and destroys their little fire of life, exactly as the strong wind which fans the big flame bigger, extinguishes a small candle beyond possibility of its being relighted. In aged and weak people the embers of life's fire require to be carefully "nursed." That is the note of the present brief discourse.

It has been said before in these pages that food can kill and food can cure: it has also been repeatedly affirmed that of all the weapons in the doctor's armamentarium, food is the chief. Compared with food the most indispensable drug which the laboratory of the chemist ever produced is insignificant. The man of forty who wishes to be his own physician must first learn the rudiments of personal physic; and the rudiments deal with food. When he has learnt these rudiments thoroughly and intelligently, he need not concern himself much about more advanced physic. What little in the way of drugs he is likely to require he may safely trust the doctor to prescribe. The first lesson in the Food Delectus may very properly at the present time treat upon winter feeding.

Now, if the writer were dealing with a class of children he would begin his instruction with a question: and that question would be this: "In winter weather is it more natural to take your food cold, or to take it hot"? He would carefully notice the answers of the children, and he would have some data for dividing them into two classes from their replies. Those two classes would be "parrots" and "original thinkers." Probably most of the children would make a guess at the answer, and some of them might accidentally guess right. But one or two would reason the matter out; and they would conclude that if it were necessary to have warmer clothing, warmer bedding, and more fire in the winter than in the summer, so it might be desirable to have warmer food: and this conclusion would be quite sound. The writer was asked out to "supper" two or three weeks ago. He wondered a little why it was supper and not dinner. When supper appeared the roast beef was cold, and so was the beetroot; the claret also was cold; there were cold blancmanges and cold mince pies; and after that cold biscuits and cold cheese. The thermometer outside marked about 9 deg. of frost. The host was a man of high scientific attainments, and his wife was a clever woman and an excellent housekeeper. That cold collation evoked, at the beginning, a suppressed groan, which later, when the troubled spirit had reached the safe shelter of its own bedroom, devoloped into an unsuppressed malediction. The cold supper was heavy as lead, and almost as indigestible. The event was forgotten, however, until, about a fortnight later, when a friend of the writer's was invited to the house of a medical man; a man whose wife had been a hospital nurse. This physiological husband and wife also gave a supper instead of a dinner, and it, too, was cold "from the eggs to the apples." These two events happening within a fortnight of each other, and in the coldest winter that has been experienced for half a century, seemed to indicate that it was a not uncommon thing even for educated people to take meals in the winter which are cold from beginning to end. What to say on such a subject as this that shall be sufficiently expressive is not very apparent. A physiologist, who is not merely a book physiologist, holds up his hands in amazement! No doubt there are happy souls, or rather, souls that have happy stomachs, that can digest cold beef, cold beetroot, cold blancmange, cold claret, cold cheese, cold biscuits, and cold butter, all piled on the top of one another, even in an arctic climate, and when the ice is a foot thick on the upper reaches of the Thames. But surely these miraculous stomachs are rare! The ordinary man, and particularly the town man, should no more sit down to such a supper in a winter like the present, than he should to a supper of Wenham ice cut into little blocks and served with parsley.

It is obvious to common sense that the first condition of comfortable and digestible feeding in winter is that the food shall be hot. If anybody wants a physiological argument, here it is; no food will digest until it is raised to a temperature of about blood heat. When it is taken cold, the first thing the stomach has to do is to warm it. Many people, particularly women, have not heat enough in their bodies to warm a stomachfull of cold, crude food. The result is, that it lies there undigested, and gives the eater no comfort until it is vomited up again. When the physiologist performs artificial digestion, what processes does he go through? He cuts up his meat into little pieces, adds his digestive fluid, and places the mixture in an oven. And why? Because he knows that by so doing he will not only get his digestion performed, but performed without trouble, and within a reasonable time.

We had intended at the outset to deal with other aspects of the winter food question; but this point of eating most foods hot in the winter instead of cold seems to be so widely neglected, and is yet so fundamentally important, that we have been led to a lengthier discussion of it than we anticipated. If, however, the concentration of attention upon this one aspect of the case should result in the opening of the eyes of fifteen or twenty thousand of our readers we shall be well content to leave other parts of the subject for consideration at a future time.

